# Type 2 Diabetes Mellitus and Thoracic Aortic Aneurysm and Dissection: An Observational Population-Based Study in Spain From 2001 to 2012

**DOI:** 10.1097/MD.0000000000003618

**Published:** 2016-05-06

**Authors:** Isabel Jiménez-Trujillo, Montserrat González-Pascual, Rodrigo Jiménez-García, Valentín Hernández-Barrera, José Mª de Miguel-Yanes, Manuel Méndez-Bailón, Javier de Miguel-Diez, Miguel Ángel Salinero-Fort, Napoleón Perez-Farinos, Pilar Carrasco-Garrido, Ana López-de-Andrés

**Affiliations:** From the Preventive Medicine and Public Health Teaching and Research Unit (IJ-T, MG-P, RJ-G, VH-B, PC-G, AL-D-A), Health Sciences Faculty, Rey Juan Carlos University, Alcorcon; Medicine Department (JMM-Y, MM-B), Hospital Gregorio Marañon; Pneumology Department (JDM-D), Hospital General Universitario Gregorio Marañón, Universidad Complutense de Madrid; Dirección Técnica de Docencia e Investigación(MÁS-F), Gerencia Atención Primaria, Madrid; and Health Security Agency (NP-F), Ministry of Health. Madrid, Comunidad de Madrid, Spain.

## Abstract

To describe trends in the rates of discharge due to thoracic aortic aneurysm and dissection (TAAD) among patients with and without type 2 diabetes in Spain (2001–2012).

We used national hospital discharge data to select all of the patients who were discharged from the hospital after TAAD. We focused our analysis on patients with TAAD in the primary diagnosis field. Discharges were grouped by diabetes status (diabetic or nondiabetic). Incidence was calculated overall and stratified by diabetes status. We divided the study period into 4 periods of 3 years each. We analyzed diagnostic and surgical procedures, length of stay, and in-hospital mortality.

We identified 48,746 patients who were discharged with TAAD. The rates of discharge due to TAAD increased significantly in both diabetic patients (12.65 cases per 100,000 in 2001/2003 to 23.92 cases per 100,000 in 2010/2012) and nondiabetic patients (17.39 to 21.75, respectively). The incidence was higher among nondiabetic patients than diabetic patients in 3 of the 4 time periods.

The percentage of patients who underwent thoracic endovascular aortic repair increased in both groups, whereas the percentage of patients who underwent open repair decreased. The frequency of hospitalization increased at a higher rate among diabetic patients (incidence rate ratio 1.14, 95% confidence interval [CI] 1.07–1.20) than among nondiabetic patients (incidence rate ratio 1.08, 95% CI 1.07–1.11). The in-hospital mortality was lower in diabetic patients than in nondiabetic patients (odds ratio 0.83, 95% CI 0.69–0.99).

The incidence rates were higher in nondiabetic patients. Hospitalizations seemed to increase at a higher rate among diabetic patients. Diabetic patients had a significantly lower mortality, possibly because of earlier diagnoses, and improved and more readily available treatments.

## INTRODUCTION

The incidence of diabetes and aortic disease has increased in recent years in both sexes.^1^ Diabetes is a major risk factor for peripheral vascular disease, coronary heart disease, and cerebrovascular disease, although it is associated with a decreased risk of progression and rupture of abdominal aortic aneurysm (AAA).^2^ A recent study showed that the incidence rates of AAA were lower in patients with type 2 diabetes mellitus (T2DM) than in those without diabetes in Spain between 2003 and 2012.^3^

Although thoracic and abdominal aortic diseases have different clinical profiles,^4^ studies have associated diabetes with a decreased rate of hospitalization from thoracic aortic aneurysm and dissection (TAAD).^5,6^ A recent study based on the Nationwide Inpatient Sample reported that the average rate of hospital discharge for TAAD among diabetic patients was 9.7 per 10,000 discharges compared with 15.6 per 10,000 discharges among nondiabetic patients.^5^ Landenhed et al^6^ investigated evidence from a community screening of 30,412 individuals participating in the Malmö Diet and Cancer Survey, and concluded that diabetes was not significantly associated with thoracic aortic aneurysm (TAA).

Since its introduction in 2005, thoracic endovascular aortic repair (TEVAR) has been increasingly used.^7,8^ TEVAR is an alternative to open surgical repair (OSR), particularly in patients for whom OSR poses a considerable risk because of coexisting medical conditions, including diabetes.^9^ Diabetes is not associated with significantly worse major outcomes after TAAD repair. Desai et al^9^ reported that diabetes was not associated with an increased risk of mortality after TEVAR (Hazard Ratio 1.1, 95% confidence interval [CI] 0.7–2.3).

Secular trends in the incidence of AAA and the use of open and endovascular AAA repair among patients with and without T2DM have been examined.^3^ However, to the best of our knowledge, no previous studies have investigated national trends in the incidence of TAAD in people with diabetes in Spain.

In this study, we used national hospital discharge data to examine trends in the incidence of TAAD among hospitalized patients with and without T2DM between 2001 and 2012 in Spain. In particular, we analyzed patient comorbidities, diagnostic and surgical procedures, and in-hospital outcomes such as in-hospital mortality (IHM) and length of hospital stay (LOHS).

## METHODS

We performed a retrospective, observational study using the Spanish National Hospital Database (“Conjunto Minimo Básico de Datos” [CMBD]), which is managed by the Spanish Ministry of Health, Social Services, and Equality, and compiles all public and private hospital data, covering more than 95% of hospital discharges.^10^ The CMBD includes patient variables (sex and date of birth), admission date, discharge date, up to 14 discharge diagnoses, and up to 20 procedures performed during the hospital stay. The Spanish Ministry of Health, Social Services, and Equality sets standards for record keeping and performs periodic audits of the database.^10^ We analyzed data collected between January 1, 2001 and December 31, 2012.

The criteria for disease and procedure were defined according to the International Classification of Diseases, Ninth Revision, Clinical Modification (ICD-9-CM), which is used in the Spanish CMBD.

We selected discharges for patients whose medical diagnosis included TAAD coded according to the ICD-9-CM as 441.01, 441.1, and 441.2 in any diagnosis field. For the purposes of this study, we focused our analysis on patients with TAAD in the primary diagnosis field. Only patients aged 50 years and older were included to minimize the number of patients with connective tissue disorders, as described by von Allmen et al.^11^

Discharges were grouped by diabetes status as follows: type 2 diabetes (ICD-9-CM codes: 250.x0 and 250.x2) and no diabetes in any diagnosis position. Patients with type 1 diabetes (ICD-9-MC codes: 250.x1 and 250.x3) were excluded.

The clinical characteristics included information on overall comorbidities at the time of diagnosis, which was assessed by calculating the Charlson Comorbidity Index (CCI). The index applies to different disease categories, the scores of which are added to obtain an overall score for each patient.^12^ We divided the patients into 3 categories: low CCI (patients with no previously recorded disease or with one disease category), medium CCI (patients with 2 categories), and high CCI (patients with 3 or more disease categories). To calculate the CCI, we used all disease categories, excluding diabetes, as described by Thomsen et al.^13^

Risk factors considered in the data analysis included smoking (ICD-9-CM codes: 305 and V1582), hypertension (401.0–405.99), and obesity (278.xx) in any diagnosis field during hospitalization due to TAAD.

We selected the following diagnostic procedures: computerized tomography (CT) of the thorax (ICD-9-CM code: 87.41 and 87.42) and cardiac ultrasound (88.72 and 88.73).

We identified OSR using the ICD-9-CM codes 38.35 and 38.45 and TEVAR using the code 39.73.

The mean LOHS and the proportion of patients who died during admission (IHM) were also estimated for each year studied.

Before the analysis, we checked the database for any missing data on the following variables: sex, date of birth, admission date, discharge date, and death during hospitalization. If any of these variables were missing, the record was removed from the analysis. Because all of the databases undergo quality control at the Ministry of Health before being sent to the investigators, we only had to exclude <0.1% of the records.

### Statistical Analysis

To assess time trends, the rates of discharge after treatment for TAAD in patients with type 2 diabetes and in nondiabetic patients were calculated per 100,000 inhabitants. We divided the study period (2001–2012) into 4 periods of 3 consecutive years each. We calculated diabetes-specific incidence rates by dividing the number of cases per year, sex, and age group by the corresponding number of people in that population group using the age and sex-adjusted estimated prevalence of diabetes obtained from National Health Surveys conducted in 2003/2004, 2006/2007, 2009/2010, and 2011/2012 and data from the Di@bet.es Study.^14,15^ We also calculated the age and sex-specific incidence rates for nondiabetic patients during each of the 3-year periods by dividing the number of cases per year, sex, and age group by the corresponding number of people in that population group (excluding those with type 2 diabetes), according to data from the Spanish National Institute of Statistics, as reported on December 31 of each year.^16^

A descriptive statistical analysis was performed for all of the continuous variables and categories by stratifying discharges for TAAD according to diabetes status. The variables are expressed as proportions or as the means with standard deviations. A bivariate analysis of variables according to year was performed using the chi-square test for linear trends (proportions) or an analysis of variance (ANOVA), as appropriate.

To test the time trend for the incidence of TAAD (as primary diagnosis or in any diagnosis field), we fitted separate Poisson regression models for patients with and without type 2 diabetes, using the year of discharge, sex, age, CCI, risk factors, diagnostic procedures, and type of repair as independent variables. A global model including the same variables and diabetes status was also fitted to assess the adjusted effect of diabetes on incidence.

For the IHM, logistic regression analyses were performed with mortality as a binary outcome using the independent variables included in the Poisson models for diabetic and nondiabetic patients and for the entire population to assess the influence of diabetes on IHM. The statistical analyses were performed using Stata version 10.1 (Stata, College Station, TX). Statistical significance was set at *P* < 0.05 (2-tailed).

### Ethical Aspects

Data confidentiality was maintained at all times in accordance with Spanish legislation. Patient identifiers were deleted before the database was provided to the authors to maintain patient anonymity. It is not possible to identify the patients on an individual level, either in this article or in the database. Given the anonymous and mandatory nature of the dataset, it was not necessary to obtain informed consent. The study protocol was approved by the Ethics Committee of the Universidad Rey Juan Carlos.

## RESULTS

We identified a total of 48,746 discharges of patients who were admitted with TAAD (any diagnosis field). TAAD was in the primary diagnosis field for 11,594 (23.78%) patients. Of these, 1103 patients (9.51%) had T2DM and 10,491 patients (90.49%) were nondiabetic.

The cumulative incidence of discharges after TAAD in the primary diagnosis field among patients with diabetes increased significantly, from 12.65 cases per 100,000 inhabitants in 2001/2003 to 23.92 patients per 100,000 inhabitants in 2010/2012. In patients without diabetes, the incidence increased significantly from 17.39 cases per 100,000 inhabitants in 2001/2003 to 21.75 patients per 100,000 inhabitants in 2010/2012. The incidence was higher in nondiabetic patients than in diabetic patients in 3 of the 4 time periods analyzed (20.26 vs 17.52 cases in 2004/2006 and 21.8 vs 17.25 cases in 2007/2009).

Table 1 shows the clinical characteristics and risk factors for hospital discharges due to TAAD in the primary diagnosis field according to diabetes status from 2001 to 2012. The mean age of the patients with diabetes was 69.46 years (SD 8.66 years), and 75.52% of the patients with diabetes were men. The mean age was lower in nondiabetic patients (68.68 years; SD 9.36 years), and 74.59% of the nondiabetic patients were men. The diabetic patients had significantly higher CCI values than the nondiabetic patients (43.43 vs 36.46%, respectively, with 2 or more coexisting conditions).

**TABLE 1:**
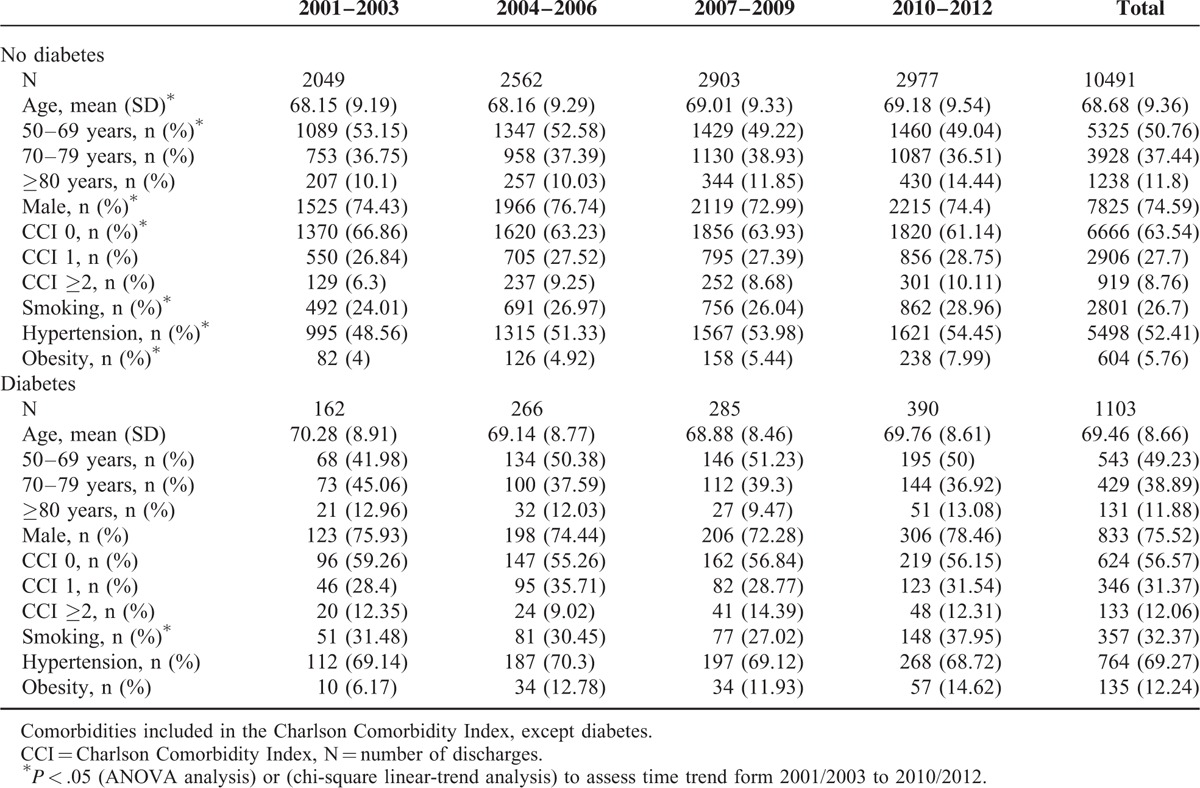
Clinical Characteristics and Risk Factors for Hospital Discharge in Patients With Thoracic Aortic Aneurysm or Thoracic Aortic Dissection as the Primary Diagnosis (Patients With and Without Type 2 Diabetes in Spain, 2001–2012)

We found that 32.37% of the diabetic patients and 26.7% of the nondiabetic patients smoked (*P* < 0.01). Hypertension and obesity were significantly more prevalent in the diabetic patients than in the nondiabetic patients (69.27% and 12.24% vs 52.41% and 5.76%, respectively).

The prevalence of smoking increased significantly in both groups during the study (Table 1).

Table 2 shows the diagnostic and therapeutic procedures and the in-hospital outcomes for patients with TAAD as the primary diagnosis according to diabetes status during the study period.

**TABLE 2:**
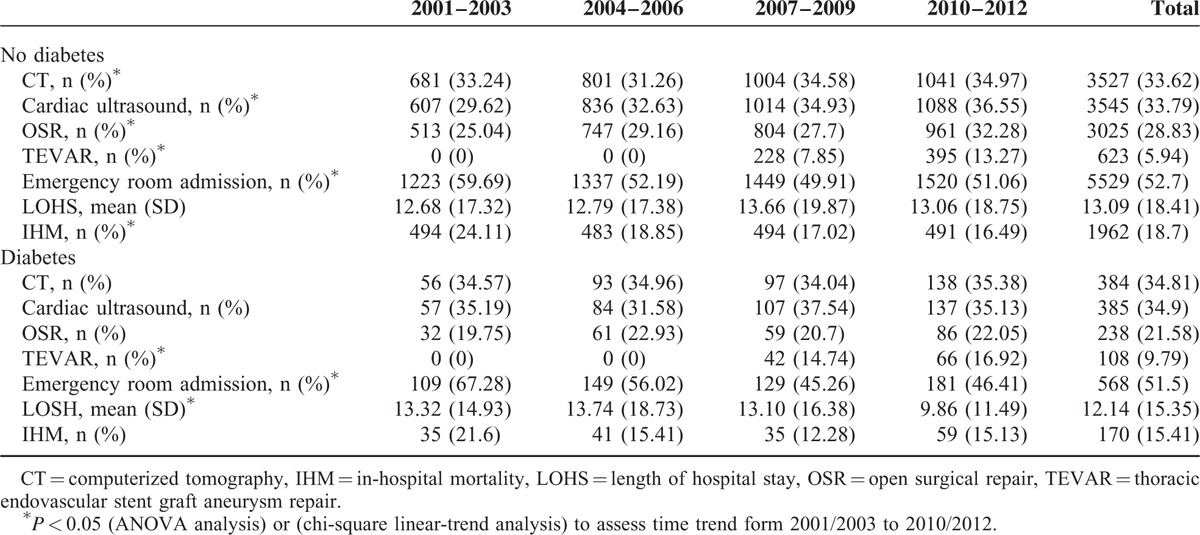
Diagnostic and Therapeutic Procedures and Outcomes of Hospital Discharges in Patients With Thoracic Aortic Aneurysm or Thoracic Aortic Dissection as the Primary Diagnosis (Patients With and Without Type 2 Diabetes in Spain, 2001–2012)

Approximately one-third of the patients with TAAD underwent CT and cardiac ultrasound.

A total of 3263 OSR procedures (7.29% [n = 238] in patients with type 2 diabetes) and 731 endovascular TAAD repair procedures (14.77% [n = 108] in patients with type 2 diabetes) were performed. OSR was more frequent in diabetic than in nondiabetic patients throughout the study period (28.83% vs 21.58%) and in each time period analyzed. However, TEVAR was more frequent in diabetic than in nondiabetic patients overall (9.79% vs 5.94%) (Table 2). We found that TEVAR was significantly more frequent in both groups during the periods analyzed (14.74% in diabetic patients and 7.85% in nondiabetic patients in 2007/2009 to 16.92% in diabetic patients and 13.27% in nondiabetic patients in 2010/2012).

Admissions to the emergency room decreased in both groups during the study (Table 2). Among diabetic patients, admissions decreased from 67.28% in 2001/2003 to 46.41% in 2010/2012.

The mean (SD) LOHS was 12.14 (15.35) days in diabetic patients and 13.09 (18.41) days in nondiabetic patients. The mean LOHS fell significantly from 13.32 days in 2001/2003 to 9.86 days in 2010/2012 in diabetic patients, but it remained stable throughout the study period in nondiabetic patients.

The IHM was 15.41% for diabetic patients and 18.70% for nondiabetic patients (*P* < 0.01). The IHM among diabetic patients did not change significantly during the study period, ranging from 21.6% in 2001 to 15.13% in 2012. However, the crude IHM decreased significantly in nondiabetic patients (24.11% in 2001/203 to 16.49% in 2010/2012).

The Poisson regression models that were constructed to compare the adjusted time trends in the incidence of discharge for patients with TAAD from 2001 to 2012 yielded an adjusted incidence rate ratio (IRR) of 0.57 (95% CI 0.53–0.60) for patients with type 2 diabetes as a primary diagnosis of TAAD when those without diabetes were used as a reference category. In other words, the adjusted incidence of discharges as the primary diagnosis of TAAD over the entire period was 0.56 times lower among nondiabetic patients. When we constructed the regression model with TAAD in any field of the database, we obtained an IRR of 0.75 (95% CI 0.73–0.77).

However, from 2001 to 2012, the adjusted IRR of having a diagnosis of TAAD on discharge in a primary field was 1.14 (95% CI 1.07–1.20) for diabetic patients; that is, the adjusted incidence increased by 14% from 2001 to 2012. The equivalent figure for nondiabetic patients was 1.08 (95% CI 1.07–1.11). These values suggest that hospitalizations were more frequent among diabetic patients than among nondiabetic patients.

For a discharge diagnosis of TAAD in any field, the IRR was higher in the diabetic patients than in the nondiabetic patients (1.37 [95% CI 1.33–1.40] vs 1.25 [95% CI 1.24–1.26]).

Table 3 summarizes the results of the multivariate analysis of trends and factors associated with IHM in diabetic and nondiabetic patients hospitalized with TAAD. Among the diabetic patients, the IHM was significantly greater in those with more comorbidities (odds ratio [OR] 1.58, 95% CI 1.06–2.35 for 1 comorbidity; and OR 2.32, 95% CI 1.40–3.84 for ≥2 comorbidities).

**TABLE 3:**
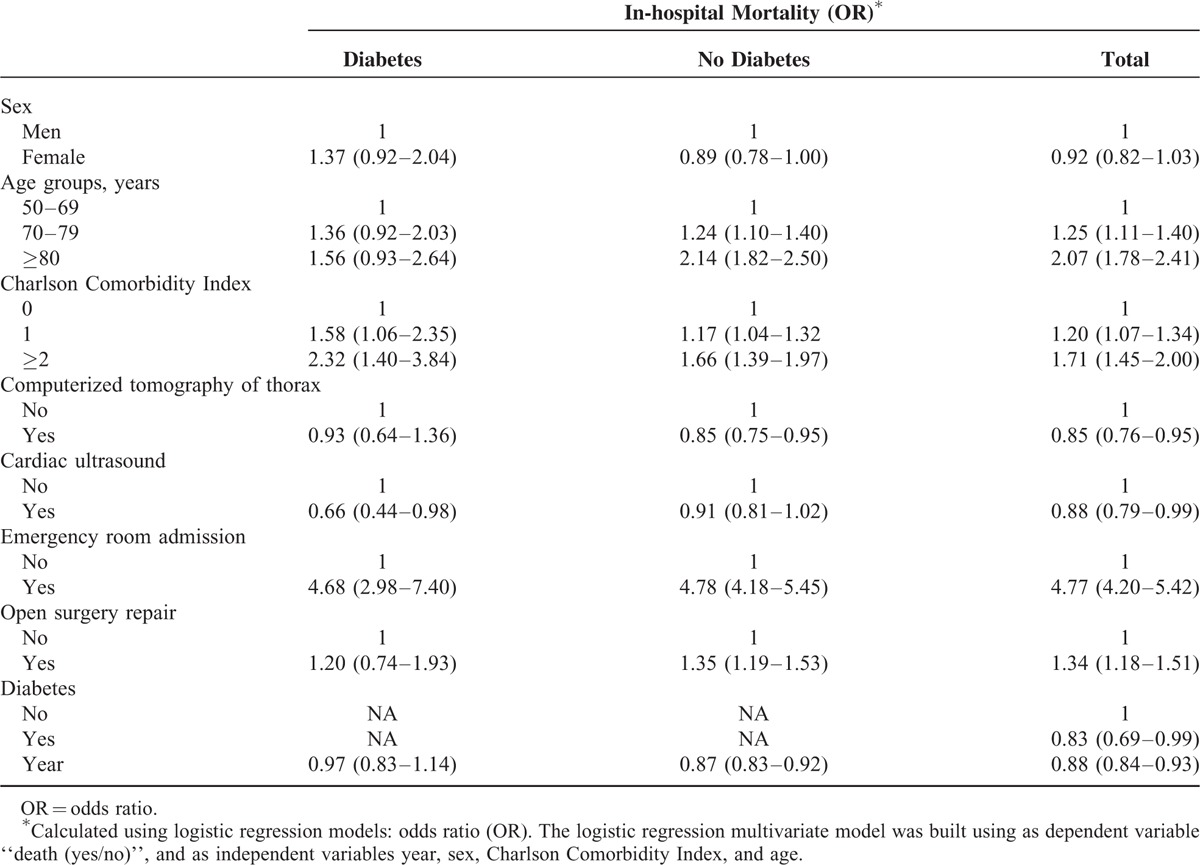
Multivariate Analysis of the Factors Associated With Mortality After Thoracic Aortic Aneurysm or Thoracic Aortic Dissection as the Primary Diagnosis (Patients With And Without Type 2 Diabetes in Spain, 2001–2012)

The diabetic patients who underwent cardiac ultrasound procedures had a 0.66-fold (95% CI 0.44–0.98) lower probability of dying during their stay than those who did not undergo these procedures.

Over the entire study period, a diabetic patient who was hospitalized after admission to the emergency room was 4.68 (95% CI 2.98–7.40) times more likely to die than a diabetic patient who was admitted electively.

The time-trend analysis failed to show a decrease in mortality from 2001 to 2012 in diabetic patients (OR 0.97, 95% CI 0.83–1.14). However, the time-trend analysis showed a significant decrease in mortality from 2001 to 2012 in nondiabetic patients (OR 0.87, 95% CI 0.83–0.92).

As shown in Table 3, for the diabetic patients, the IHM was significantly higher in older persons, in those with more comorbidities, and in those who had been admitted to the emergency room.

The patients without diabetes who had a CT scan had a 0.85-fold (95% CI 0.75–0.95) lower probability of dying during their stay than those who did not undergo this procedure.

When we analyzed the entire database, we found that mortality was significantly lower among diabetic patients than nondiabetic patients after adjusting for all of the covariates (OR 0.83, 95% CI 0.69–0.99).

## DISCUSSION

Using data from the Spanish National Hospital Discharge Database, we found that the rates of discharge for TAAD in patients with and without type 2 diabetes increased significantly from 2001 to 2012. Using the Swedish national healthcare registers from 1987 to 2002, Olsson et al^17^ found that discharge rates increased over time due to improved diagnostic techniques and case ascertainment.

In our study, incidence rates were higher in nondiabetic patients in 3 of the 4 time periods studied, although hypertension and smoking were more prevalent in diabetic patients than in nondiabetic patients. Previous studies found lower rates of aneurysm and dissection in diabetic patients.^5,18^ In a national case-control study conducted by Prakash et al^5^ in the USA, the authors concluded that hospitalization due to TAAD was significantly less common among diabetic patients than among nondiabetic patients. They suggested that a direct vascular effect of hyperglycemia on the aortic wall could inhibit the progression of TAAD.^5^ After adjusting for age and sex, and analyzing TAAD as the primary diagnosis or a diagnosis in any field of the database, we found that hospitalization was increasingly more frequent among diabetic patients than among nondiabetic patients. Possible explanations include improvements in diagnostic techniques that enable TAAD to be identified and diagnosed earlier and at a less severe stage.^5^

We found an increase in the frequency of the TEVAR procedure in both groups. The procedure was more frequent in diabetic patients. The authors of a large nationwide population-based study of aneurysm and aortic dissection in England and Wales reported an increase in the incidence of thoracic aortic dissection from 7.2 per 100,000 inhabitants in 1999 to 8.8 per 100,000 inhabitants in 2010, and an increase in the incidence of TAA from 4.4 per 100,000 inhabitants in 1999 to 9.0 per 100,000 inhabitants in 2010. The authors concluded that this increase in incidence was associated with an increase in the use of aortic repair, particularly TEVAR.^11^ Patients undergoing repair more recently had a higher frequency of comorbidities, including diabetes. In the USA, Kilic et al^19^ indicated that annual rates of repair for both intact and ruptured TAA increased significantly during the study period (intact 2.2–10.6 per 1 million; ruptured 0.8–1.3 per 1 million; *P* < 0.05), primarily as a result of increases in the frequency of endovascular repair in recent years.

Mortality was significantly lower in diabetic patients than in nondiabetic patients: the fact that diabetes is a well-known risk factor for vascular disease could have directed physicians toward more targeted therapy for TAAD.^5^ A recent revision in the management of acute aortic dissection indicated that mortality is highest during the first 48 hours after symptom onset, and concluded that the time from diagnosis to treatment was significantly associated with a history of previous cardiovascular episodes.^20^

The “obesity paradox” has also been postulated as an explanation for lower morbidity in diabetic patients.^21^ Given that obesity is much more frequent among diabetic patients, a high body mass index (BMI) may be associated with a decrease in mortality because of TAAD. A recent study concluded that BMI was negatively associated with aortic aneurysm disease.^22^

We found that patients who underwent OSR were more likely to die during their stay than those who did not. Our results are consistent with those reported in the literature, indicating higher perioperative mortality for OSR.^23,24^ In 2011, Goodney et al^23^ found that perioperative mortality was lower in patients undergoing TEVAR than in patients undergoing OSR for both intact TAA (6.1 vs 7.1%; *P* < 0.05) and ruptured TAA (28 vs 46%; *P* < 0.01). In the USA, Hughes et al^8^ compared the results of OSR with those of TEVAR and reported that, after adjustment for preoperative comorbidities, including the presence of diabetes, the likelihood of death was reduced by 46% among patients undergoing endovascular repair compared with OSR (OR 0.54; *P* < 0.01). In 2008, Echeverria et al^25^ evaluated the effect of TEVAR on death and found that their results agreed with those of other studies showing a beneficial effect of TEVAR in acute aortic disease. The authors concluded that the increased use of this procedure will likely lead to continuous improvement in survival in the future.

We found a significant decrease in admission to the emergency room in both groups. Further investigations are required to clarify this observation.

As expected, the IHM was significantly greater in patients admitted to the emergency room from both groups.

Smoking and hypertension are well-known risk factors for aortic diseases including aortic dissection and aneurysm.^26^ Diabetes is very frequently associated with obesity, and this last disease is directly related to blood pressure (BP) values.^26–29^ The prevalence of hypertension among diabetic patients is significantly higher than that among age and sex-matched nondiabetic patients, which indicates that it is particularly relevant to screen diabetic patients for aortic diseases.^26,27,30^

Several considerations regarding the role of hypertension and physical exercise in our results require discussion. The ICD-9-CM includes codes 401 to 405 for hypertensive disease. Among these codes, “401,” which corresponds to “essential hypertension,” represents over 90% of the diagnoses in our population, with similar figures for those with and without diabetes. The CMBD database does not include systolic or diastolic measurements; therefore, it is not possible to determine BP classification.

In our study, the prevalence of hypertension for diabetic patients was approximately 70%, which is lower than the figure reported for patients in primary care centers in our country (80%–90%).^29,31,32^

Therefore, the prevalence of hypertension we reported is surely underestimated because, among those hospitalized patients who achieved good BP control with pharmacological treatment, the code for essential hypertension may not be recorded in their discharge report. It is estimated that only approximately 20% to 30% of T2DM patients reach the BP objective in Spain.^29,31–33^

Furthermore, previous investigations have found that patients with diabetes have a higher prevalence of masked hypertension than patients without diabetes,^34,35^ and it has been found that patients with T2DM have a greater propensity for exercise hypertension.^36–38^

Mancia et al showed that masked hypertension is associated with an increased risk of mortality and future development of sustained hypertension, and this may explain the increased cardiovascular risk associated with a hypertensive response to exercise (HRE) at moderate exercise workloads.^39^

Additionally, increased levels of serum cholesterol and insulin resistance have been shown to be positively correlated with changes in BP with exercise, but not at rest. These metabolic impairments may hinder vascular reactivity during exercise and increase vascular resistance, also leading to an HRE. Physical fitness may also be an important factor because it is related to insulin resistance and exercise BP responses.^40–42^

Unfortunately, the Spanish National Hospital Discharge Database does not include information on physical activity; therefore, it is not possible to analyze this variable. Previous studies conducted in Spain using other sources have found that a significantly lower proportion of people with diabetes engage in physical activity than age and sex-matched nondiabetic patients. Therefore, the effect of physical activity on masked hypertension is expected to be small.^27,28,30,43^

Clinical trials have found that supervised exercise (3 times per week for 6 months, compared with general advice about physical activity) improved fitness and body composition, but there were no reductions in BP among T2DM patients. The lack of change in arterial stiffness suggests resistance to exercise-induced BP reduction in persons with T2DM.^44^

Based on the previous data, we believe that masked and exercise-induced hypertension may play a role in thoracic aneurisms among diabetic patients, and this topic requires further investigation.

The strengths of our findings lie in the large sample size, the 12-year follow-up period, and the standardized methodology, which has been used to investigate diabetes and its complications in Spain and elsewhere.^3^

### Limitations of the Study

Nevertheless, our study is subject to several limitations. The data about the physical activity and occupations are missed. Our data source was the CMBD, an administrative database that contains discharge data for hospitalizations in Spain and uses information the physician has included in the discharge report. Therefore, our findings are limited by the lack of anatomical data; for example, we do not know the size of the aneurysms that were treated.

Other studies have identified factors that may influence aortic dimensions and that were not included in our investigation because these variables were not collected in the Spanish Hospital Discharge Database. These factors include, among others, having a sedentary occupation^45^ and body size (height, weight, and body surface area). Height is particularly relevant because it is also associated with Marfan syndrome.^26,46^

Also, additionally, we cannot discriminate between the various forms of thoracic aneurysm and dissection (proximal aneurysm, acute type A dissection, acute type B dissection, and chronic type A and B dissection). The mortality rate was lower in diabetic patients, although this may be because diabetic patients were less likely to suffer acute type A dissection, which is the most lethal aortic condition.^4,6,17^

Another significant limitation is the fact that we did not classify diabetic patients into groups based on the therapy used to control blood glucose, with the result that we were unable to provide data on the control of blood glucose before or after surgery.

The database is limited by its anonymity (no identifying items such as clinical history number), which makes it impossible to detect whether the same patient was admitted more than once during the same year. In addition, patients who moved from one hospital to another could appear twice. Nevertheless, this dataset, which was introduced in Spain in 1982, is a mandatory register, and its coverage is estimated to be greater than 95%.^10^

Concerns have been raised about the accuracy of routinely collected datasets; however, these datasets are periodically audited. Consequently, the quality and validity of our dataset has been assessed and shown to be useful for health research.^47^

Furthermore, previous studies comparing hospital discharge data with other data sources have found that a high degree of concordance was detected for diabetes mellitus and for hypertension, particularly regarding the age and sex-specific distribution patterns.^48,49^

## CONCLUSIONS

In conclusion, Spanish national data show that rates of discharge for TAAD in patients with and without type 2 diabetes increased significantly from 2001 to 2012, although the incidence rates were higher in nondiabetic patients in 3 of the time periods studied. The frequency of hospitalization seems to be increasing at a higher rate among diabetic patients than among nondiabetic patients.

Mortality was significantly lower among diabetic patients. Admission to the emergency room was associated with higher IHM in both groups.

We found a decrease in the use of OSR and an increase in the use of TEVAR in patients from both groups. Patients who underwent an OSR procedure were more likely to die during their stay than those who did not undergo this procedure.

Given the rapid increase in the prevalence of diabetes and the aging of the population, our findings emphasize the need for further improvement in the control of risk factors for TAAD in people with diabetes. Future studies are necessary to monitor changes and explore the causes underlying the trends found in this study in more depth.
