# Type 2 Diabetes Mellitus and Thoracic Aortic Aneurysm and Dissection: Erratum

**DOI:** 10.1097/01.md.0000494759.72626.c0

**Published:** 2016-08-26

In the article “Type 2 Diabetes Mellitus and Thoracic Aortic Aneurysm and Dissection”,^1^ which appeared in Volume 95, Issue 18 of *Medicine*, funding information was not provided in full in the original article. The article has since been corrected online.
